# Malignant peritoneal mesothelioma presenting with bilateral hydronephrosis and renal insufficiency: a case report and literature review

**DOI:** 10.3389/fsurg.2024.1342657

**Published:** 2024-04-24

**Authors:** Jisheng Luo, Jinhong Pan, Chuanhua Zhong, Lina Wang, Heng Zhang

**Affiliations:** ^1^ Department of Urology, Guiqian International General Hospital, Guiyang, China; ^2^ Department of Pathology, Guiqian International General Hospital, Guiyang, China

**Keywords:** peritoneal mesothelioma, hydronephrosis, percutaneous nephrostomy, ureteral stent, case report

## Abstract

**Introduction:**

Malignant peritoneal mesothelioma (MPM) is an extremely rare tumor with nonspecific clinical manifestations, making diagnosis challenging.

**Case presentation:**

Herein, we report a case of MPM with occult onset presenting with bilateral hydronephrosis and renal insufficiency. A 30-year-old man was admitted to the Urology Department because of recurrent bilateral lower back pain. The etiology was unclear after a series of laboratory tests, imaging examinations, bone marrow aspiration, renal puncture biopsy, ascites examination, ureteroscopy, and so on. Finally, MPM was diagnosed by laparoscopic exploration and biopsy. Moreover, during the course of the disease, the patient's bilateral ureters were compressed, and the obstruction could not be relieved after the placement of ordinary ureteral stents. Percutaneous nephrostomy or metal ureteral stenosis was appropriate in managing malignant ureteral obstruction as it could improve renal function.

**Conclusions:**

The onset of this case was insidious, and the diagnosis was difficult, with a poor prognosis. To date, only a handful of cases have been reported. We hope this case can provide some enlightenment for our clinical work.

## Introduction

Malignant peritoneal mesothelioma (MPM) is an aggressive neoplasm that can rapidly spread within the abdominal area. Patients with peritoneal mesothelioma usually present with an abdominal mass, ascites, and abdominal pain. Herein, we report a case with bilateral hydronephrosis and renal insufficiency as the first symptoms. The case was extremely challenging to diagnose, which was finally confirmed based on laparoscopic exploration and biopsy. This study aimed to provide clinical evidence for the diagnosis, treatment, and prognosis of rare MPM.

## Case presentation

**Figure 1:**
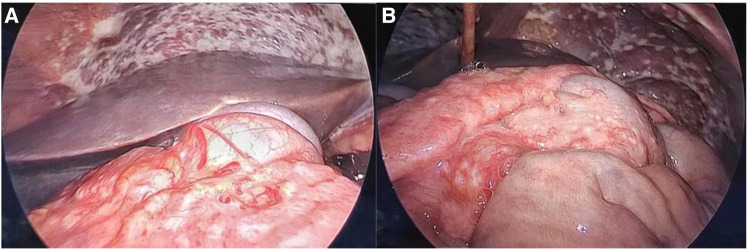
(**A**) Numerous small nodules carpeting all visualized peritoneal and some parts of intestinal tube surfaces. (**B**) Tumor infiltrating the entire omentum and forming a large, firm mass.

**Figure 2:**
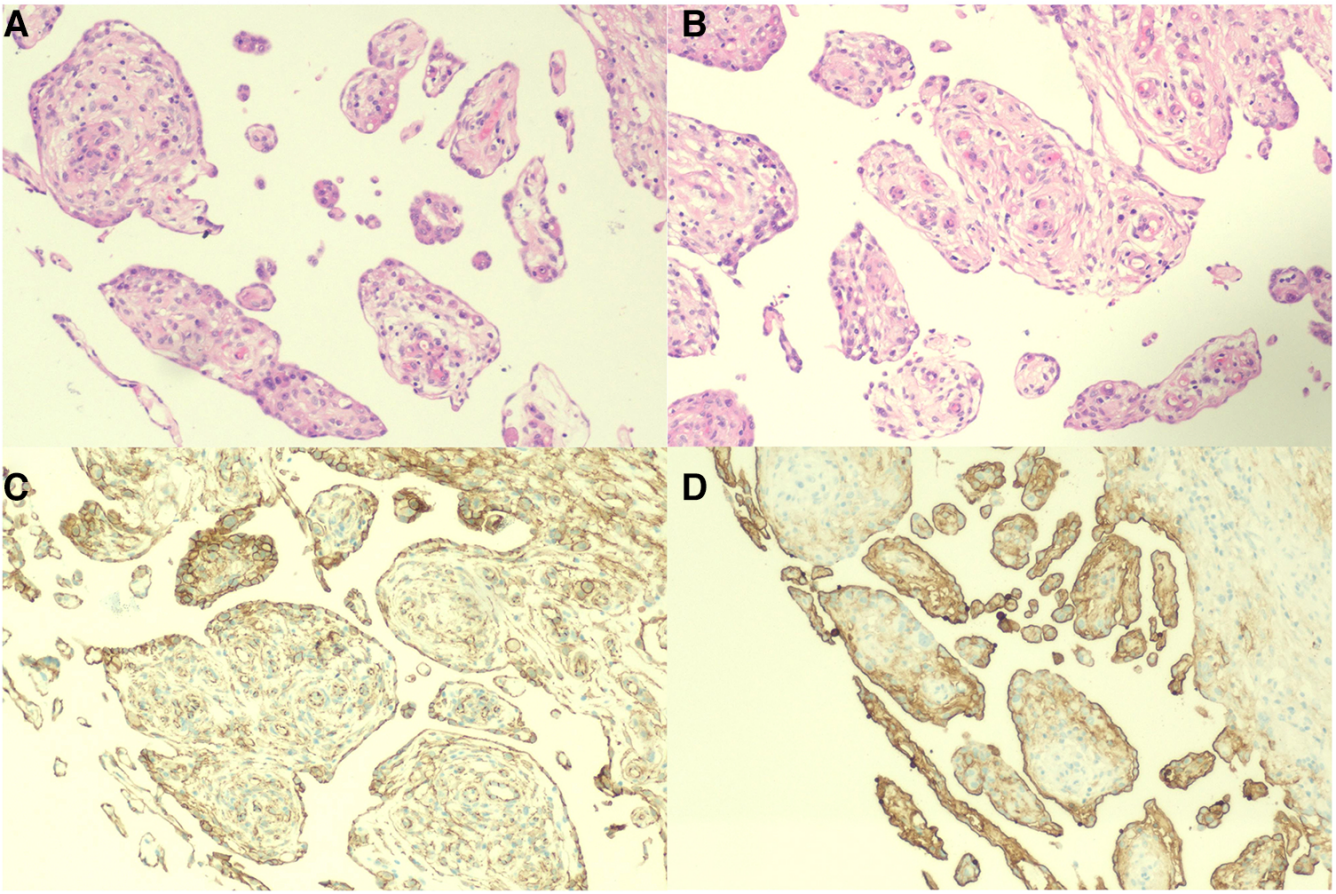
Histology and immunohistochemistry of the omentum. (**A**,**B**) Mass of cells lined with a papillary structure of monolayer flat/cuboidal mesothelial cells with a milder nucleus and acidophilic cytoplasm (H&E, ×100). (**C**,**D**) Immunohistochemistry results for calretinin and MC (HBME-1) (×100).

A 30-year-old man was admitted to the Urology Department because of repeated bilateral lower back pain for 3 months. At the onset of the illness, computed tomography (CT) scans revealed bilateral hydronephrosis, and the serum creatinine (SCr) level was 123 μmol/L. Symptoms improved after the placement of bilateral ureteral stents. However, the back pain recurred 1 month later, accompanied by abdominal distension, nausea, and vomiting. Enhanced CT scans revealed bilateral hydronephrosis, splenomegaly, and ascites. Hydronephrosis had aggravated, with SCr levels increasing to 305 µmol/L. A transurethral cystoscopy revealed no lower urinary tract obstruction. Ureteroscopy indicated narrowing of the lower part of the bilateral ureter, approximately 6 cm from the ureteral opening. During hospitalization, the patient's condition continued to deteriorate, with a progressive decrease in urine volume. The highest SCr level was 470 μmol/L. A plain CT scan revealed bilateral hydronephrosis, splenomegaly, abdominal effusion, peritoneal thickening with exudative changes, and intestinal wall thickening, with no significant mass observed. We performed a bilateral percutaneous nephrostomy (PCN) and abdominal catheterization. Ascites analysis revealed no definite malignant cells. Serum IgG4 level testing yielded negative results. A renal needle biopsy indicated moderate to severe chronic tubulointerstitial injury. Finally, laparoscopic exploration was performed, and intraoperative findings revealed numerous small, shiny, whitish nodules carpeting all visualized peritoneal surfaces and some parts of the intestinal tubes. The tumor infiltrated the entire omentum, forming a large, firm mass (Figure 1). A biopsy of the omentum itself confirmed the diagnosis of peritoneal mesothelioma. Histological findings revealed fibrous tissue hyperplasia with inflammatory cell infiltration and papillary hyperplasia of the overlying mesothelium. The immunohistochemical (IHC) markers were as follows: calretinin (+), CK (+), desmin (-), HMB45 (-), Ki-67 (10%+), CD34 (-), MC (HBME-1) (+), melan-A (-), and B-catenin (+) (Figure 2).

Unfortunately, the patient refused to undergo therapy and chose to be discharged for recuperation. During the follow-up, he died at home 1 month after discharge.

## Discussion and conclusions

Malignant peritoneal mesothelioma is an aggressive neoplasm of the serosal membranes, first reported by Miller and Wynn in 1908 (1). It accounts for approximately 25% of malignant mesotheliomas. Asbestos exposure is a well-recognized high-risk pathogenic factor for MPM (2). The patient was a deaf–mute construction worker with no clear history of asbestos exposure. Patients with peritoneal mesothelioma usually present with abdominal pain, distention, ascites, and an abdominal mass. Hydronephrosis rarely occurs as the first symptom. We searched PubMed for articles addressing peritoneal mesothelioma and hydronephrosis and found five relevant articles, screened by title and abstract. Two articles were finally included in the review. Detailed case data of four patients were obtained (Table 1).

**Table 1:** Characteristics of four cases of peritoneal mesothelioma presenting with hydronephrosis, as screened from PubMed and our patient.

Ref.	Age (years)	Sex	Chief complaint	Diameter of the tumor	Hydronephrosis	Ascites	Diagnosis	Drainage	Therapy	Prognosis
Yoshida et al. (3)	39	M	Right back pain	8 cm	Right	Without	Tumor excision	No description	No description	No description
Yoshida et al. (3)	50	M	Right lower abdominal pain	No description	Bilateral	No description	Biopsy	No description	No description	No description
Yoshida et al. (3)	43	F	General fatigue, nausea	No apparent mass	Bilateral	Without	Biopsy (percutaneous needle biopsy)	Ureteral stenting	Chemotherapy (gemcitabine, methotrexate)	Died (9 months)
McCaffrey et al. (4)	59	F	Left iliac fossa pain	A left iliac fossa mass	Bilateral	Massive	Pelvic biopsy, benign cystic peritoneum mesothelioma	Ureteral stenting	Exploratory laparotomy, conservative	Follow-up (6 years)
This patient	30	M	Bilateral back pain	No apparent mass	Bilateral	Massive	Biopsy (laparoscopic exploration)	Ureteral stenting, PCN	Conservative	Died (5 months)

Among these cases, the majority had a survival period of only a few months after the first diagnosis. Four out of five presented with combined bilateral hydronephrosis. Ureteral stenting was initially used in the majority of patients to drain hydronephrosis. Renal function improvement was observed in two patients. Therefore, the drainage mode should be considered when dealing with ureteral obstruction. There is no consensus on the optimal method for malignant ureteral obstruction (MUO). Some studies (5) showed that the postoperative stent failure rate of extrinsic malignant ureteral obstruction was 42%–45%. However, there was no difference in median survival compared with PCN. Metallic stents and some new material stents provide more options. The type and level of obstruction, renal insufficiency, degree of hydroneurosis, and length of obstruction >3 cm have been identified as predictors of stent failure in MUO patients. Accordingly, all these aspects should be taken into consideration. Clinician preference and patient comfort are also important factors.

Diagnosing MPM is extremely challenging, as it is characterized by irregular or nodular peritoneal or mesenteric thickening, omental mass, and ascites in imaging evaluations. The accuracy of ascitic cytology is only 50% (6). Laparoscopy is superior to CT in the evaluation of localized peritoneal metastases. The gold standard for MPM diagnosis is pathological examination. Morphologically, there are mainly three subtypes of MPM: epithelioid, sarcomatoid, and biphasic. Epithelioid MPM is the most common and least aggressive subtype, accounting for approximately 60% of cases. Our patient belongs to the epithelioid type, as evidenced by the histological findings showing papillary hyperplasia of the overlying mesothelium. However, the diffused growth pattern of the tumor and the limited tissue obtained by laparoscopic biopsy present challenges for pathological examination. In combination with the prognosis of the patient, it can be mixed with a few sarcomatoid types. IHC staining is the most valuable and feasible method for the differential diagnosis of MPM (7). The recognized positive markers are calretinin, CK 5/6, WT-1, HBME-1, thrombomodulin, podoplanin, mesothelin, and D2-40, while the negative markers are TTF1, carcinoembryonic antigen (CEA), Ber Ep4, B72.3, MOC31, and CD15. It is recommended to use at least two positive and two negative markers for differential diagnosis (8).

In 2011, Yan et al. (9) proposed a TNM staging system based on the extent of peritoneal disease burden (T), intra-abdominal nodal metastasis (N), and extra-abdominal metastasis (M) to standardize and guide the clinical treatment and prognostic evaluation of MPM. The T stage is determined by calculating the peritoneal carcinomatosis index (PCI). This assessment combines lesion size (0–3) with tumor distribution (abdominopelvic and mesenteric regions 0–13) to quantify the extent of disease as a numerical score (PCI-0 to 39). The 5-year survival rates of stage I, II, and III patients were reported to be 87%, 53%, and 29%, respectively. The patient’s PCI was >30, categorizing MPM as stage III (T4). This staging was an independent prognostic factor for malignant peritoneal mesothelioma.

Cytoreductive surgery (CRS) combined with hyperthermic intraperitoneal chemotherapy (HIPEC) is the initial preferred treatment for MPM. An analysis by the US HIPEC Collaborative, including 130 patients with MPM (all histologic subtypes included) who underwent CRS-HIPEC, reported a 5-year OS of 67.8% and a conditional OS of 89.7%. The strongest predictor of long-term survival is complete cytoreduction (10). Our patient presented with diffuse MPM, with extensive peritoneal, small bowel serosal, or mesentery involvement, which is not amenable to complete cytoreduction. The proposed treatment plan is intravenous chemotherapy and HIPEC, and immunosuppressive therapy can be added if physical conditions allow. The current preferred intraperitoneal chemotherapy is cisplatin alone or in combination with cisplatin/doxorubicin, the most commonly used combination therapy. Systemic therapy is an alternative treatment for inoperable patients. The International Expanded Access Program assessed pemetrexed regimens for 109 patients with MPM. Patients received pemetrexed, pemetrexed plus cisplatin, or pemetrexed plus carboplatin as either first-line or second-line therapy. For patients who received pemetrexed plus cisplatin, the 1-year survival rate was 57.4%. For patients who received pemetrexed alone, the median survival rate was 10.3 months, and the 1-year survival rate was 41.5%. Survival rates are not available for pemetrexed plus carboplatin (11). In recent years, molecular therapy and immunotherapy have attracted increasing attention. BAP1, TP53, NF2, and ALK, which are commonly mutated genes in MPM (12), are expected to become potential therapeutic targets. A phase II study evaluated the activity of pembrolizumab in 64 mesothelioma patients who had been treated with one or two chemotherapy regimens. Among these patients, only eight (12.5%) had MPM, and they exhibited lower overall response rates than patients with pleural mesothelioma in the study. Whether PDL1 expression is predictive of benefit remains unknown. In another cohort study evaluating the clinical efficacy of immunotherapy in patients with advanced MPM, the overall response rate was 19%, with a reported median progression-free survival (PFS) of 5.5 months (13)*.* Immunotherapy is an exciting future direction for MPM, and we look forward to the results of more clinical trials.

Peritoneal mesothelioma with bilateral hydronephrosis as the first symptom is rare and the diagnosis can be confirmed by laparoscopic exploration biopsy when necessary. Because of severe ureteral compression, PCN, or metal stents with high tension, offer more advantages than ordinary stents in improving renal function.

## Data Availability

The original contributions presented in the study are included in the article/Supplementary Material, further inquiries can be directed to the corresponding author.
